# Classifying Autism Spectrum Disorder Using the Temporal Statistics of Resting-State Functional MRI Data With 3D Convolutional Neural Networks

**DOI:** 10.3389/fpsyt.2020.00440

**Published:** 2020-05-15

**Authors:** Rajat Mani Thomas, Selene Gallo, Leonardo Cerliani, Paul Zhutovsky, Ahmed El-Gazzar, Guido van Wingen

**Affiliations:** Department of Psychiatry, Amsterdam University Medical Center, Amsterdam, Netherlands

**Keywords:** neuroimaging, 3D convolutional neural network, classification, autism, Autism Brain Imaging Data Exchange, deep learning

## Abstract

Resting-state functional magnetic resonance imaging (rs-fMRI) data are 4-dimensional volumes (3-space + 1-time) that have been posited to reflect the underlying mechanisms of information exchange between brain regions, thus making it an attractive modality to develop diagnostic biomarkers of brain dysfunction. The enormous success of deep learning in computer vision has sparked recent interest in applying deep learning in neuroimaging. But the dimensionality of rs-fMRI data is too high (~20 M), making it difficult to meaningfully process the data in its raw form for deep learning experiments. It is currently not clear how the data should be engineered to optimally extract the time information, and whether combining different representations of time could provide better results. In this paper, we explored various transformations that retain the full spatial resolution by summarizing the temporal dimension of the rs-fMRI data, therefore making it possible to train a full three-dimensional convolutional neural network (3D-CNN) even on a moderately sized [~2,000 from Autism Brain Imaging Data Exchange (ABIDE)-I and II] data set. These transformations summarize the activity in each voxel of the rs-fMRI or that of the voxel and its neighbors to a single number. For each brain volume, we calculated regional homogeneity, the amplitude of low-frequency fluctuations, the fractional amplitude of low-frequency fluctuations, degree centrality, eigenvector centrality, local functional connectivity density, entropy, voxel-mirrored homotopic connectivity, and auto-correlation lag. We trained the 3D-CNN on a publically available autism dataset to classify the rs-fMRI images as being from individuals with autism spectrum disorder (ASD) or from healthy controls (CON) at an individual level. We attained results competitive on this task for a combined ABIDE-I and II datasets of ~66%. When all summary measures were combined the result was still only as good as that of the best single measure which was regional homogeneity (ReHo). In addition, we also applied the support vector machine (SVM) algorithm on the same dataset and achieved comparable results, suggesting that 3D-CNNs could not learn additional information from these temporal transformations that were more useful to differentiate ASD from CON.

## Introduction

Neuroimaging holds the promise of objective diagnosis and prognosis in psychiatry. But unlike neurological disorders, psychiatric disorders do not show obvious structural brain abnormalities. Researchers have long posited that brain abnormalities of psychiatric patients are particularly reflected in functional scans such as resting-state functional magnetic resonance imaging (rs-fMRI) (1). These scans involve mapping the blood oxygenation level (a proxy for brain activity) throughout the brain every 1 or 2 s—resulting in a 4-D data product, 3-D of the brain, and 1-D in time. Typically, at a scanning resolution of 4 mm and 300 temporal sampling points, this results in a 20 million dimensional feature vector. Hidden somewhere in this high-dimensional spatio-temporal signal are the biomarkers that could distinguish between healthy and psychiatric subjects. Mining these rs-fMRI images for such patterns is the challenge we are facing.

Machine learning (ML) approaches in recent years have demonstrated enormous potential in processing images and videos. Deep learning, in particular, has been extremely successful in myriad different fields (2). Apart from algorithmic improvements, deep learning has been successful largely due to the massive datasets available in the image processing fields. Unfortunately, this is not the case in neuroimaging. Large datasets are scarce or non-existent. But some initiatives like the Autism Brain Imaging Data Exchange (ABIDE) have tried to aggregate brain imaging datasets of individuals with autism spectrum disorder (ASD) and healthy controls (CON) from various sites around the world to build up a reasonably sized dataset. The complete dataset including the first and second wave of the data aggregation (ABIDE-I/ABIDE-II) comprises around 2,100 subjects including ASD and CON. Albeit the availability of such a database, the dimensionality of the input data is still too large to be used without any preprocessing or feature engineering.

Since rs-fMRI data contains a spatio-temporal signal, different approaches have been tried to reduce its dimensionality. Primarily, this can be categorized as approaches that try to reduce dimensionality in space or in time. One approach for spatial down-scaling is the use of brain atlases. In this approach, about one million voxels in space are locally averaged in about 100 to 400 different brain regions depending on the atlas, thus considerably reducing the dimension of the input data. These 100 to 400 averaged time courses are then either used in a 1-D convolutional network (3) or are first used to calculate a cross-correlation matrix which subsequently is used as a feature in various machine learning methods (4, 5). For example, (4) reached an accuracy of 70% on ABIDE-I using the cross-correlation matrix on time courses extracted using the CC400 atlas.

In the neuroimaging literature, there are many examples of reducing the dimensionality of brain volumes by summarizing the time domain, while maintaining intact the 3D spatial dimensions of the data. These different methods extract different aspects of the time series. For example, the amplitude of low-frequency fluctuation (ALFF) is a measure that is posited to reveal differences in the underlying processing of the brain, and is calculated as the ratio of spectral power in two distinct frequency ranges and has been linked to atypical development in previous studies (6, 7). This measure is calculated independently for each voxel. Other measures instead are calculated for each voxel but carry information about distant or neighboring connectivity. This is the case for regional homogeneity (ReHo), which is the temporal coherence or synchronization of the BOLD time series within a set of a given voxel's nearest neighbors, and of voxel-mirrored homotopic connectivity (VMHC), which is the synchrony in patterns of spontaneous activity between homotopic (geometrically corresponding) regions in each hemisphere. The complete list of summary measures considered in this work is described in *Resting-State Functional MRI Summary Measures*.

We aimed to retain the full spatial resolution as input by summarizing the temporal dimension per voxel as a single number. Most of the summary measures we employed were informed by the neuroimaging literature. To the best of our knowledge, no previous study attempted an extensive use of such measures using deep learning algorithms. Moreover, since each summary measure extracts different aspects of the temporal dimension of the rs-fMRI data, we further aimed to combine the different inputs for classification.

First, we used each of the nine single summary measures (single-measure model or SM-models) independently as input to a 3-D convolutional neural network (3D-CNN). Then, inspired by the concept of the *wisdom of the crowd* and *weak learners*, which refers to the fact that single models that perform weakly individually could perform better when combined, we tested this by combining the nine summary measures. To do so, we explored two different strategies: the multi-measure ensemble (MM-ensemble), and the creation of a single model, called multi-measure-model (MM-model). In the MM-ensemble approach, we independently trained models on each of the summary measures and allowed each model to *vote* toward the final classification outcome of the test sample. The class with the majority vote was then used as the predicted outcome. In the MM-model, we combined the 3D summary measure volumes for each subject and input them as channels to a single 3D-CNN model. Since there is a debate whether a deep learning model can perform better than a linear or other conventional machine-learning models in neuroimaging (8, 9), and in order to form a robust baseline, we also performed the classification using a linear-SVM. To gain insight into the information the models are using for the classification task, we performed an *occlusion experiment*. This type of analysis describes which regions of the brain contribute maximally to the output of the most successful neural networks. The code used of all the experiments performed is available on GitHub^1^.

## Materials and Methods

### Autism Brain Imaging Data Exchange Dataset

We used the ABIDE dataset for all of our experiments. The ABIDE I+II datasets is a collection of structural (T1w) and functional (rs-fMRI) brain images aggregated across 29 institutions (10), available for download^2^. It includes 1,028 participants with a diagnosis of autism, Asperger or pervasive developmental disorder-not otherwise specified (called ASD from now on), and 1,141 typically developing participants (CON). Virtually all the ASD participants were high functioning (99.95% with IQ > 70), most of the included participants were adolescents (median age 13 years, range between 5 and 64 years of age), 1/3 of whom were diagnosed as ASD, and 20% of the total participants were female, which represents an important addition with respect to most previous autism studies which focused on males exclusively. The rs-fMRI image acquisition time ranges from 2 to 10 min, with 85% of the datasets meeting the suggested duration (~ 4–5 min) for obtaining robust rsfMRI estimates (11). We chose to cut off the minimum scan duration to 100-time points, which led us to include 96% of the whole ABIDE I+II dataset (N=2,085, N(ASD)=993), the vast majority of which (95%) with a minimum acquisition time of 4 min.

### Resting-State Functional MRI Preprocessing

The preprocessing was done using the Configurable Pipeline for the Analysis of Connectomes (C-PAC, (12). We followed a preprocessing strategy adopted by the Preprocessed Connectome Project initiative^3^. This will allow others to replicate and extend the findings in this paper. Our preprocessing pipeline, consisted of i) motion correction, ii) nuisance regression which included head motion modeled as 24-regressors (13), scanner drift modeled using a quadratic and linear term, and physiological noise modeled using the five principal components from a decomposition of white matter and cerebrospinal fluid voxel time series (CompCor) (14), iii) coregistration of the resulting rs-fMRI image to the subject's anatomical image using FMRIB Software library (FSL) Boundary-Based (BB) register. Finally, iv) the images were normalized onto the standard Montreal Neurological Institute (MNI) space (4 mm) with the non-linear registration algorithm from Ants (15). All the above steps were configured using C-PAC's singularity image^4^.

### Quality Check and Subject Selection

After preprocessing, we selected subjects from the ABIDE I+II data-sets following the list provided in (16); the authors performed an automatic quality control (QC) by selecting those subjects that retained at least 100 frames or 4 min of fMRI scans after motion scrubbing based on framewise displacement. Then subjects were visually inspected by the authors (16). Only subjects that passed the entire QC process are included in this study. The procedure yielded a total of 1,162 subjects, 620 of which were classified as ASD (773 from ABIDE-I and 389 from ABIDE-II). Please refer to the original article for an extensive description of the procedure. See **Table 1** for more details on the sample composition.

**Table 1:** Sample composition.

	Site	Group	Group # (females #)	Mean age	s.d. age	Selected for leave-site-out CV
Abide II	ABIDEII-KKI_1	CON	26 (6)	10.49	1.51	Yes
		ASD	88 (34)	10.36	1.20	
Abide II	ABIDEII-NYU_1	CON	45 (4)	10.25	5.87	No
		ASD	29 (2)	9.52	3.38	
Abide II	ABIDEII-OHSU_1	CON	22 (3)	11.55	2.22	Yes
		ASD	42 (21)	10.45	1.68	
Abide II	ABIDEII-SDSU_1	CON	32 (7)	13.06	3.19	No
		ASD	24(2)	13.07	2.96	
Abide II	ABIDEII-TCD_1	CON	14 (0)	15.29	3.54	No
		ASD	18 (0)	16.31	2.76	
Abide II	ABIDEII-UCLA_1	CON	9 (0)	11.99	1.76	No
		ASD	9 (4)	9.88	2.12	
Abide II	ABIDEII-USM_1	CON	15 (2)	19.16	6.94	No
		ASD	16 (3)	23.98	7.80	
Abide I	CALTECH	CON	1 (0)	20.20	–	No
		ASD	2 (1)	38.50	25.03	
Abide I	CMU	CON	1 (0)	33.00	–	No
		ASD	2 (0)	27.00	8.49	
Abide I	KKI	CON	20 (4)	10.02	1.45	No
		ASD	28 (8)	10.01	1.16	
Abide I	LEUVEN_1	CON	14 (0)	21.86	4.11	No
		ASD	14 (0)	23.00	2.83	
Abide I	LEUVEN_2	ASD	6 (3)	14.82	1.25	No
Abide I	MAX_MUN	CON	7 (0)	12.86	7.31	No
		ASD	4 (0)	14.00	6.16	
Abide I	NYU	CON	69 (10)	14.86	7.28	Yes
		ASD	90 (26)	15.32	6.04	
Abide I	OHSU	CON	12 (0)	11.43	2.18	No
		ASD	13 (0)	10.24	1.02	
Abide I	OLIN	CON	18 (3)	16.39	3.47	No
		ASD	13 (2)	16.85	3.85	
Abide I	PITT	CON	9 (0)	18.70	7.11	No
		ASD	4 (2)	24.91	8.11	
Abide I	SBL	CON	1 (0)	35.00	–	No
Abide I	SDSU	CON	12 (1)	14.79	1.82	No
		ASD	21 (6)	14.11	1.87	
Abide I	STANFORD	CON	14 (3)	9.61	1.48	No
		ASD	19 (4)	9.99	1.64	
Abide I	TRINITY	CON	21 (0)	16.51	2.92	No
		ASD	25 (0)	17.08	3.77	
Abide I	UCLA_1	CON	41 (6)	13.10	2.62	Yes
		ASD	28 (4)	13.23	2.03	
Abide I	UCLA_2	CON	9 (0)	12.67	1.56	No
		ASD	4 (2)	12.73	0.62	
Abide I	UM_1	CON	46 (8)	12.80	2.37	Yes
		ASD	50 (17)	13.95	3.14	
Abide I	UM_2	CON	13 (1)	14.88	1.55	No
		ASD	21 (1)	16.72	3.97	
Abide I	USM	CON	43 (0)	23.84	8.47	No
		ASD	23 (0)	20.55	8.29	
Abide I	YALE	CON	28 (8)	12.75	3.05	No
		ASD	27 (0)	12.83	2.69	

The table illustrates the sample composition: the site of data collection in alphabetical order (SITE_ID), the number of subjects categorized as ASD or CON (group #) and the number of female individuals in each group (females #), mean, and standard deviation of age per group (in years, respectively mean, age, and s.d. age). The last column describes if data contained in the specific site was used as test-data in the leave-site-out procedure.

### Resting-State Functional MRI Summary Measures

The preprocessed rs-fMRI images were transformed into nine *summary measures* that reduced the temporal dimension of the data to a single number per voxel by highlighting different statistical features of the time series. The summary measures chosen were:

- **Regional homogeneity (ReHo)**, a voxel-based measure of brain activity which evaluates the similarity or synchronization between the time series of a given voxel and its nearest neighbors (17).
- **Amplitude of low frequency fluctuations (ALFF)**, defined as the total power within the frequency range between 0.01 and 0.1 Hz, and thus indexes the strength or intensity of low frequency oscillations (18, 19).
- **Fractional amplitude of low frequency fluctuations (fALFF)**, defined as the power within the low-frequency range (0.01–0.1 Hz) divided by the total power in the entire detectable frequency range, representing the relative contribution of specific low frequency oscillations to the whole frequency range (20).
- **Degree centrality (DC, weighted)**, is a measure of local network connectivity and identifies the most connected nodes by counting the number of direct connections (edges) to all other nodes. As such, a node with high DC will have direct connections to many other nodes in the network. Degree centrality analysis tends to emphasize higher order cortical association areas while showing reduced sensitivity for paralimbic and subcortical regions (20, 21).
- **Eigenvector centrality (EC, weighted)** is a measure of global network connectivity. The EC of a given node reflects the number of direct connections it has with other nodes that have high centrality (21).
- **Local functional connectivity density (LFCD, weighted)**, a quantification of the number of local and global functional connections for each voxel in the brain (22, 23)
- **Entropy**, a measure of organization and predictability of a system (17).
- **Voxel-mirrored homotopic connectivity (VMHC)**, a quantification of functional homotopy by providing a voxel-wise measure of connectivity between hemispheres. This is done by computing the connectivity between each voxel in one hemisphere and its mirrored counterpart in the other (20, 21).
- **Auto-correlation lag**, the correlation between its past and present states; thus, a high correlation indicates that the series state does not change over time (24).

The mean and standard deviation of the summary measures were calculated within voxels across brain volumes belonging to the training sample, and these values were used to normalize the entire dataset. This procedure, referred to as feature scaling, speeds up the convergence of the model during training. Moreover, in the particular context of the ABIDE dataset, in which imaging data are known to vary in quality and have been collected using different scanner hardware and sequence, the procedure might help to mitigate the heterogeneity of the data.

### Network Architecture

In this work, we utilized a 3D CNN architecture to classify ASD from CON subjects. The architecture is inspired by (16). We reduce the number of layers and filters per layer to reduce the number of parameters to around 257 k, therefore reducing computational complexity and cost. Therefore, starting from Khosla et al.'s model, we performed a non-systematic parameter and hyper-parameter search, and carried out the full experiments on the best configuration. Before honing in on the above architecture we also experimented with 3D versions of the popular computer-vision architectures like ResNet-50 (25), Visual Geometry Group (VGG)-net (26), and it's variants without much success.

**Figure 1:**
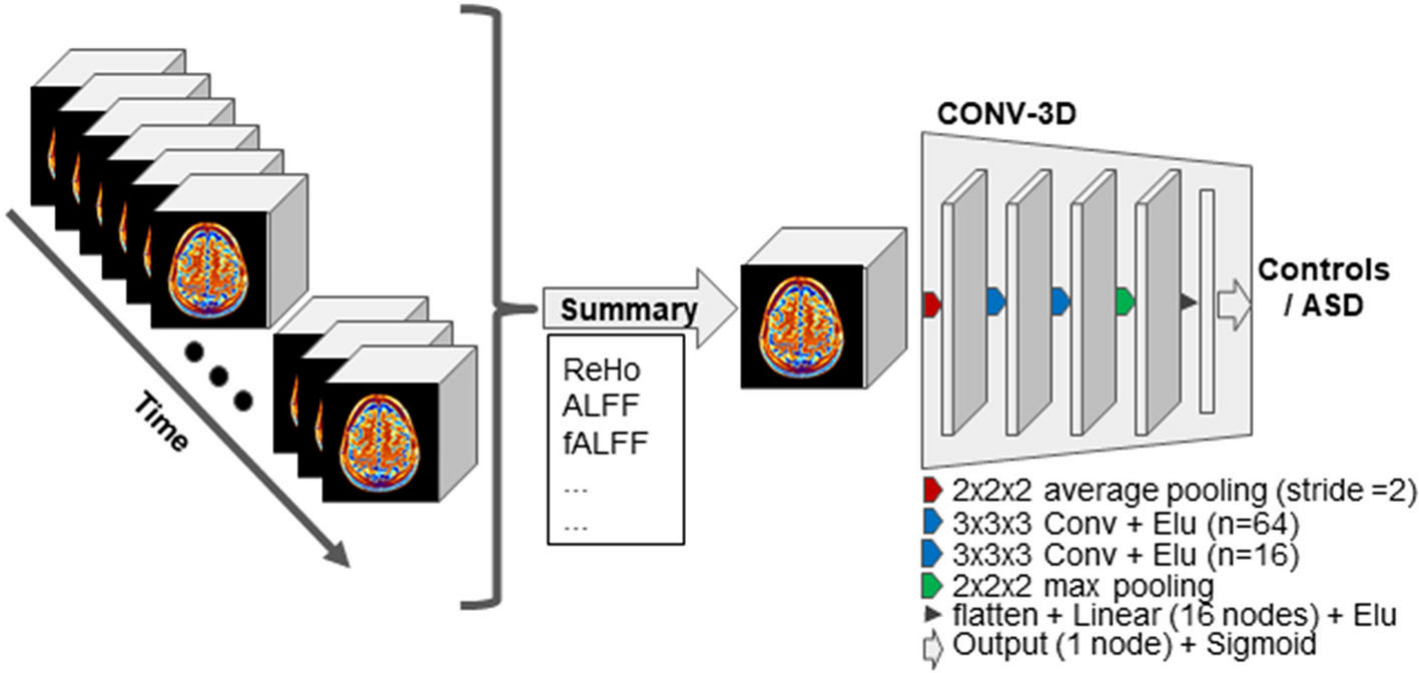
Schematic representation of our approach. First, the resting-state functional MRI (fMRI) data are summarized on the time domain while keeping the spatial resolution intact. The resulting 3D volume is then input to the 3D-convolutional neural network (CNN) models for the classification task. The 3D-CNN model used across experiments is schematically illustrated. Abbreviation: **“**Conv**”** stands for convolutional layer, and the n associated to each layer indicates the number of kernels. **“**Linear**”** stands for linear fully connected layer.

Our model (**Figure 1**) consisted in a first layer of average pooling of size 2 and stride 2, which functioned as a sort of down-sampling function. Two convolutional layers with a exponential linear unit (ELU) activation followed. The first convolutional layer had 64 filters of size 3 and the second convolutional layer had 16 filters of size 3. The convolutional layers were followed by a max-pooling layer of kernel dimension of 2. The output was flattened and fed to a first fully connected layer with 16 nodes and again ELU activation. The last layer was a fully connected layer with one node for final labels classification.

In the SM-model approach, we used the architecture described above on each of the nine rs-fMRI summary measures, resulting in nine independent models. In the MM-ensemble approach, the classification problem is first independently solved for each summary measure as for the SM-model, but the final prediction is computed as the majority *vote* of the individual binary class predictions. In the MM-model approach, the input of the 3D-CNN is formed by concatenating the summary measures. Each channel is a summary measure, therefore the input is now represented as nine-channel 3D volumes. The other architectural parameters are kept the same.

We used the linear-SVM as a baseline to compare our 3D-CNN models against. For this purpose, the volumes of each participant were first flattened into a 1D array and voxels that were part of a brain mask were used as features for the SVM. The masks were created independently for each summary measure and contained only voxels that appeared as non-zero in at least 90% of the subjects. In the SM-model approach, we trained the linear-SVM on each of the nine summary measures. In the MM-ensemble approach, the outcome of the model was defined as the class with the majority SM-models votes. To perform the MM-model, for each subject, the nine volumes representing each of the nine summary measures were flattened and concatenated in a single 1D array. The linear-SVM was then trained on the 1D arrays. In all the linear-SVM experiments we used the default parameter options in the machine learning python package Scikit-learn^5^ (27, 28).

### Cross-Validation Procedure

Cross-validation is a resampling procedure used to evaluate machine learning models on independent data for a limited data sample. For each experiment, we implemented two cross-validation schemes: i) five-fold cross-validation (CV) and ii) a leave-site-out CV procedure.

Before implementing the five-fold cross-validation schemes, the data were split into training/validation-data, consisting of the 90% of the total dataset, and test-data, consisting of the remaining 10%. The five-fold cross-validation involved randomly dividing the set of observations into groups, or folds, of approximately equal size. The first fold is treated as validation set, and the model is fit on the remaining four folds, called the training set (29). In our application, the validation set was used to select the best trained model for each fold. To do so, during training we evaluated the performance on the validation set after each epoch. The model with the best accuracy on the validation set between epochs was used for testing. The test-data are therefore invariant across CV folds. Although this is not the most common application of K-fold CV, this procedure is often used in competitions [e.g., predictive analytic challenge—PAC, 2019, see (30)], it guarantees no contamination between training/validation-data and test-data, and, importantly the test-data can be easily interpreted as a common benchmark to compare models.

For the leave-site-out CV procedure, we selected five sites with the highest number of total subjects (ASD/total), namely New York University (NYU) (90/159), Kennedy Krieger Institute - 1 (KKI-I) (88/114), University of Miami - 1 (UM-I) (50/96), University of California, Los Angeles (UCLA)-I (28/69), Oregon Health & Science University (OHSU)-I (42/64). In this method, each site was held out as the test set in turn. The rest of the data were randomly split into 90% of the set used for training and 10% used for validation. The validation set was used to select the best performing model between epochs, which was then applied to the test-site. In this way, for the leave-site-out CV we had as many test sets as sites (i.e., five).

### Occlusion of Brain Regions

We performed an occlusion experiment to assess which regions of the brain maximally determined model performance. We iterated over all the regions of the Harvard-Oxford atlas (thresholded at 25 and downsampled at 4 mm to match our data resolution) systematically by occluding that part of the cortex with a mask set to be zero and monitoring the probability of the classifier. We reran the five fold CV procedure on the test dataset for each occluded region of interest (ROI) and calculated average balanced accuracy and F1 score between folds. The drop in performance when the ROI is removed from the data compared to the original data is a suggestion of how much the voxels contained in the ROI contributed to the original results. The 10 ROIs that contributed the most, hence that showed the most substantial drops, are reported.

## Results

In our experiments we pursued the goal of classifying the rs-fMRI brain images of ASD subjects from healthy controls by means of their temporal summary measures. We assessed the potential of achieving this goal of each of our nine summary measures in independent models, the advantage of employing all the measures together in an ensemble model approach, and the use of the measures in a single model with as many channels as the summary measures as input. In the next sections, we illustrate the results obtained by the 3D-CNN models and by the linear-SVM.

### 3D-Convolutional Neural Network Results

For our 3D-CNN architecture we used an architecture very similar to that of Khosla et al. (16). Other architectures like ResNet and VGG-net did not perform very well at all and therefore we only present results from the architecture described in the *Network Architecture*. The models were trained with a mini-batch of 32, for a maximum of 50 epochs. The loss of the validation set had converged by then. The neural network weights were optimized by the binary-cross entropy loss and Stochastic gradient descendent (SGD) with learning rate of 0.001 and momentum of 0.9. The same model and parameters were used across all experiments.

**Figure 2:**
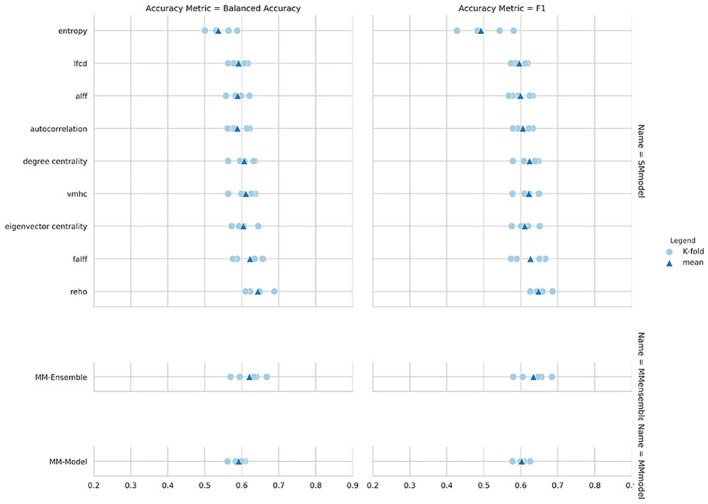
Balanced accuracy and F1-score for the nine single measure (SM)-models trained on the summary measures, and for the MM-ensemble and multi-measure (MM)-model.

**Figure 3:**
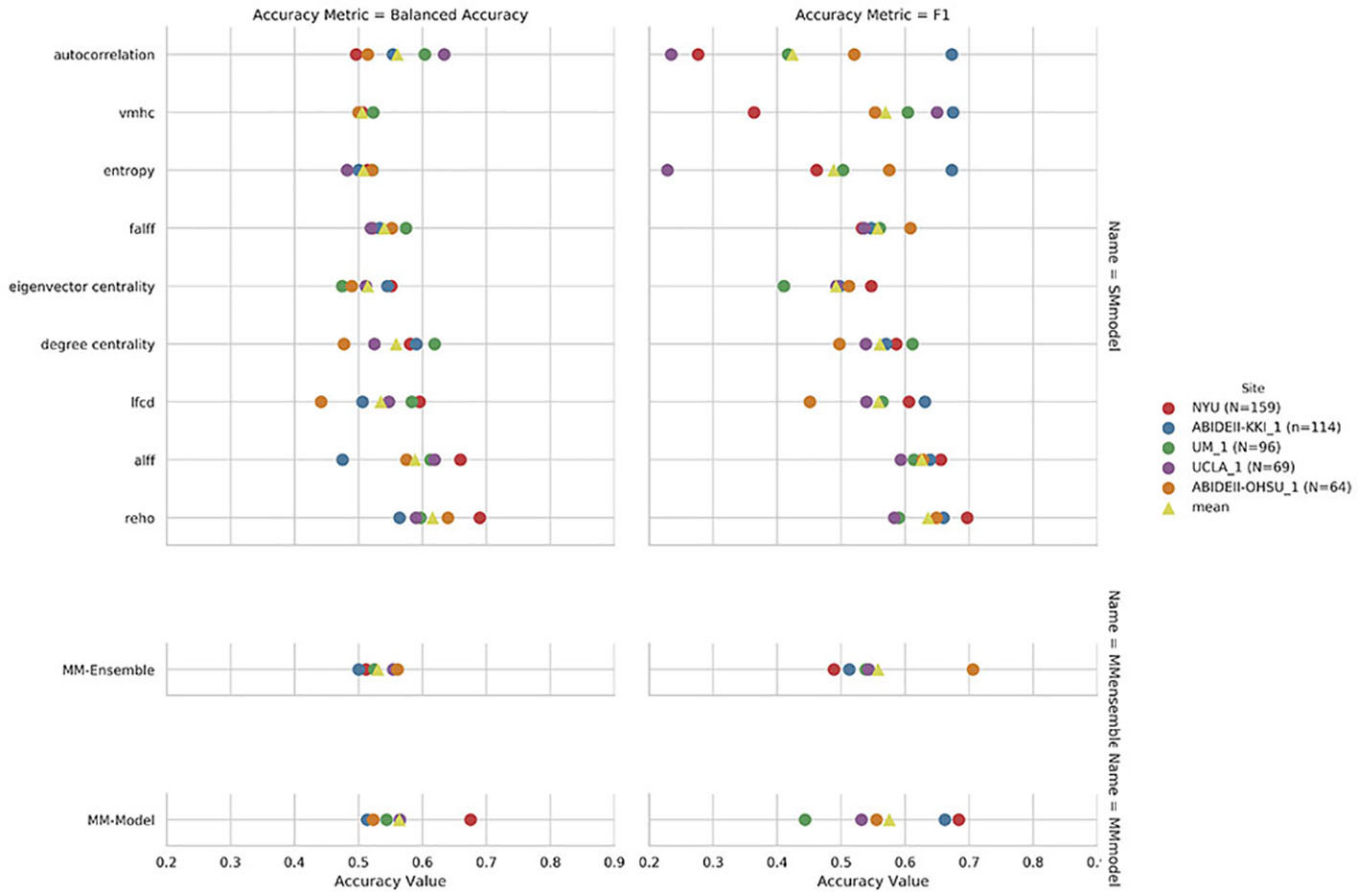
Leave-site-out balanced accuracy and F1-score for the nine single measure (SM)-models trained on the summary measures mentioned on the y axis, and for the multi-measure (MM)-ensemble and MM-model for each test-site indicated in the legend.

Regarding the variability in classification performance between models, we observed a difference between the two CV procedures. When assessed by five-fold CV procedure, all tested models performed at around 60% accuracy for the classification task (**Figure 2** and **Table 2**), with little variation between models' performances. On the contrary, the leave-site-out CV procedure shows high variability in terms of model performances with relatively good performance for the NYU site and poor performance for the other sites (**Figure 3**, **Table 3**, and **Supplementary Table S1**).

**Table 2:** Mean performance evaluated as balanced accuracy (accuracy) and F1-score obtained for 3D convolutional neural network (CNN) and linear support vector machine (SVM) with five-fold cross-validation (CV).

	3D-CNN		SVM	
	Accuracy	F1-score	Accuracy	F1-score
ReHo	**0.64**	0.65	0.66	0.66
fALFF	0.62	0.63	0.57	0.58
Degree centrality	0.61	0.62	0.63	0.64
VMHC	0.61	0.62	0.62	0.62
Eigenvector centrality	0.60	0.61	0.61	0.63
Autocorr lag	0.59	0.61	0.57	0.59
LFCD	0.59	0.60	0.63	0.65
ALFF	0.59	0.60	0.57	0.58
Entropy	0.54	0.49	0.56	0.57
MM ensemble	**0.6**4	**0.6**6	**0.66**	**0.67**
MM model	0.59	0.60	0.61	0.62

In bold = Highest score in that column.

**Table 3:** Mean performance evaluated as balanced accuracy (accuracy) and F1-score obtained for 3D convolutional neural network (CNN) and linear support vector machine (SVM) as SM-models, MM ensemble, and MM model with leave site out cross-validation (CV).

	3D-CNN		SVM	
	Accuracy	F1-score	Accuracy	F1-score
ReHo	**0.62**	**0.64**	**0.63**	**0.62**
fALFF	0.59	0.63	0.61	0.61
VMHC	0.56	0.57	0.62	0.59
Degree centrality	0.56	0.56	0.60	0.59
fALFF	0.54	0.56	0.57	0.54
LFCD	0.53	0.56	0.63	0.61
Eigenvector centrality	0.51	0.49	0.59	0.57
Entropy	0.51	0.49	0.53	0.52
Autocorr	0.51	0.42	0.54	0.55
MM ensemble	0.56	0.59	0.53	0.56
MM model	0.56	0.58	0.61	0.62

See **Figure 3** and **Supplementary Tables S1** and **S2** for details on how each test site performed.

In bold = Highest score in that column.

The overall best performing model was the MM-ensemble, which achieved balanced accuracy of 64% and F1 score of 66%, averaged over the five-folds of the CV procedure. With the leave-site-out CV procedure the performance of the MM-ensemble dropped to an average balanced accuracy of mean 56% and F1 score of 59%, suggesting that this model does not do well in generalizing to new sites. The overall lowest performance was obtained by the entropy SM-model.

Among the SM-models, the ReHo SM-model is the best performing and its classification accuracy is similar to the MM-ensemble when evaluated with five-fold CV (balanced accuracy mean: 64%, F1-score mean: 66%). Its performance remained comparable when tested using the leave-site-out CV procedure (balanced accuracy mean: 62%, F1-score mean: 64%).

**Figure 4:**
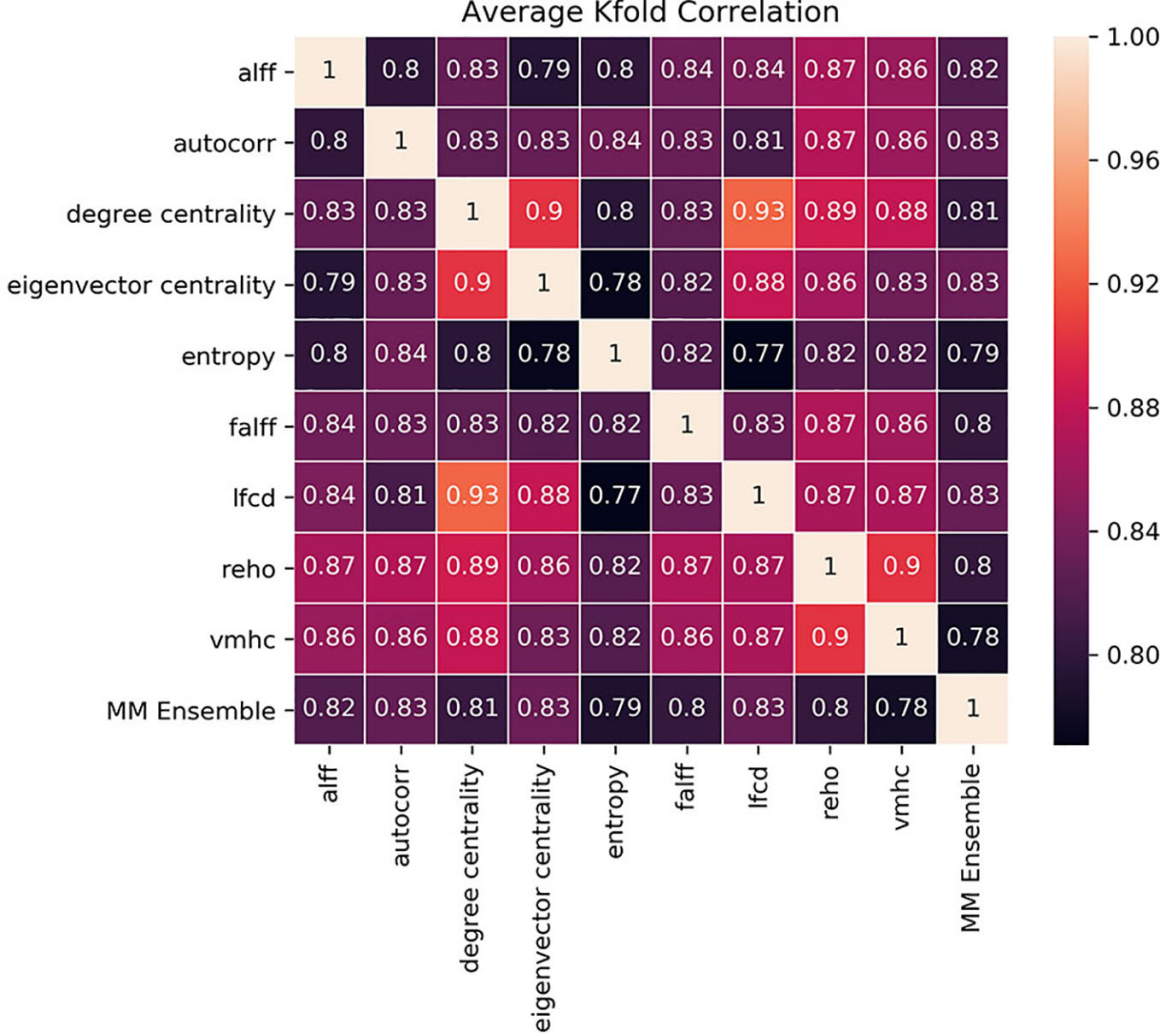
Kendall-correlations between classification predictions between models trained and tested with different summary measures (SM-models) and between each SM-models and the MM-ensemble. The last column and the last row show the correlation between the predictions of each SM-model and the MM-ensemble. The correlation is made on the common test-dataset, calculated for each K-fold separately and then averaged for visualization purposes. The color coding is the Kendall-correlation averaged between K-folds.

The idea of the ensemble strategy is to learn several different weak learners and combine them to output predictions based on the multiple predictions returned by these weak models. Therefore the success of the MM-ensemble strategy should be due to the independent contribution of the information gathered from the nine summary measures. To assess that the SM-models are processing independent information to classify the subjects, we estimated Kendall-correlations between predictions of each SM-model, separately for each fold. If the correlation matrix shows a cluster of SM-models whose predictions are highly correlated, this would suggest that the SM-models are picking up on similar patterns to classify ASD from CON and therefore they share information. To assess the contribution of each measurement to the MM-ensemble output, we estimated Kendall-correlations between each SM-model and the MM-ensemble prediction, again separately for each fold. If any of the SM-models has a large influence on the MM-ensemble, this would appear as a stronger Kendall-correlation between their predictions. The correlation results, averaged between folds, are reported in **Figure 4**. There were no discernable clusters of SM-models whose predictions aligned, and all the correlations between SM-models ranged between 0.77 and 0.90. In the same fashion, none of the predictions of the SM-models stood out for correlating with the prediction of the MM-ensemble, and all the correlations ranged between 0.77 and 0.83. The analysis illustrates that variation in the degree of correlation between predictions of SM-models is more than variation between the correlation of each SM-model and the MM-ensemble prediction. Interestingly, while the ReHo SM-model and the MM-ensemble reached a similar performance accuracy, this cannot be explained as a more substantial contribution of ReHo to the MM-ensemble, since the correlation between the two is not stronger than between MM-ensemble and other SM-models predictions. These observations indicate that the MM-ensemble benefits from independent contributions from each of the SM-model outputs.

### Linear-Support Vector Machine Results

In order to establish a baseline for the performance of the 3D-CNN models, we performed the classification task following the same procedures but now using a linear SVM. As for the 3D-CNN experiments, we implemented the two evaluation schemes: 1) five-fold cross-validation (CV) and 2) a leave-site-out CV procedure. Linear-SVM does not require a validation procedure to select the algorithm, but to be able to compare the results to the one obtained by 3D-CNN experiments, we applied exactly the same procedure described above and limited the data used to train the linear-SVM to the 90% of the training/validation dataset. The averaged balanced accuracy and F1-score for each experiment evaluated *via* five-fold and leave-site-out CV are reported in **Tables 2** and **3** respectively (see also **Supplementary Table S2** for results of single site in the leave-site-out CV).

The results of the linear-SVM confirmed the patterns identified by the 3D-CNN experiments with the linear-SVM performing the classification task at around the 60% accuracy level. The highest balanced accuracy and F1 scores were achieved by the MM-ensemble (66 and 67% respectively, five-fold CV), but it suffered when tested on new sites (balanced accuracy mean: 53%, F1-score mean: 56%). While the accuracy of the linear-SVM MM-ensemble evaluated by five-fold CV is in line with the one of the 3D-CNN counterpart, its decrease in performance when evaluated by leave-site-out CV was steeper than what was observed for the 3D-CNN MM-ensemble.

Again, the ReHo SM-model was the best performing model in comparison to other SM-models and performed comparable to the MM-ensemble when the outcome was evaluated with five-fold CV (balanced accuracy mean: 66%, F1-score mean: 66%). Interestingly, its performance remained comparable when evaluated using the leave-site-out procedure (balanced accuracy mean: 63%, F1-score mean: 62%). The lowest performance was again observed for the entropy SM-model.

### Occlusion of Brain Regions Results

**Figure 5:**
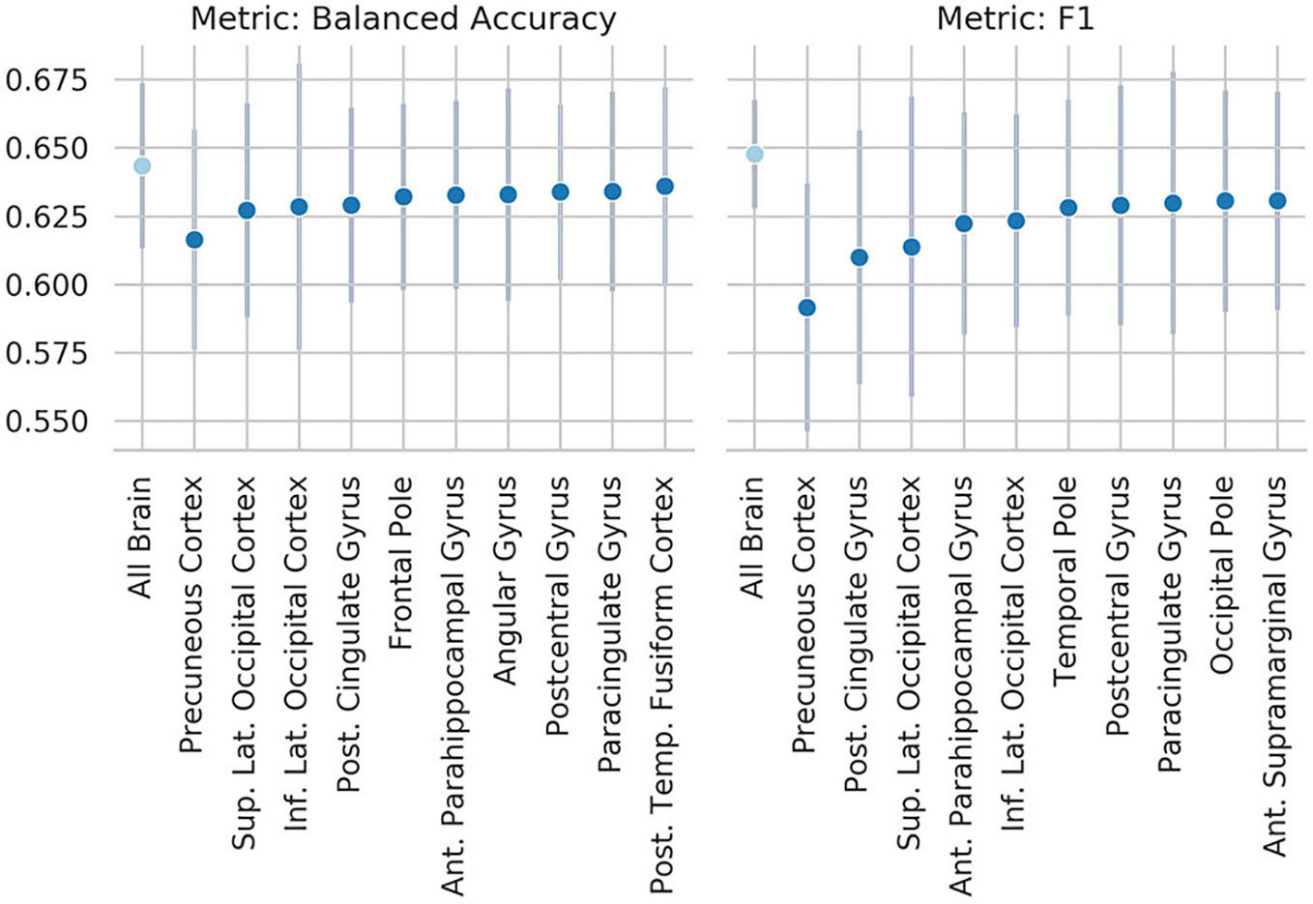
Results of the occlusion experiment for the regional homogeneity (ReHo) SM-model, balanced accuracy, and f1 are reported as mean and standard deviation across five folds. “all brain” indicates the results obtained by the original model, when all the ROIs are included. Region of interests (ROIs) have been identified using the Harvard-Oxford atlas (threshold at 25 and downsampled at 4 mm). The ROI names indicate the results when the named ROI is masked from the brain volume, therefore the drop from the “all brain” result is a suggestion of how much the voxels contained in the ROI contributed to the original results. Only the 10 ROIs that contributed the most are reported. Sup., superior portion; Inf., inferior portion; Lat., lateral portion; Post., posterior; Temp., temporal.

**Figure 6:**
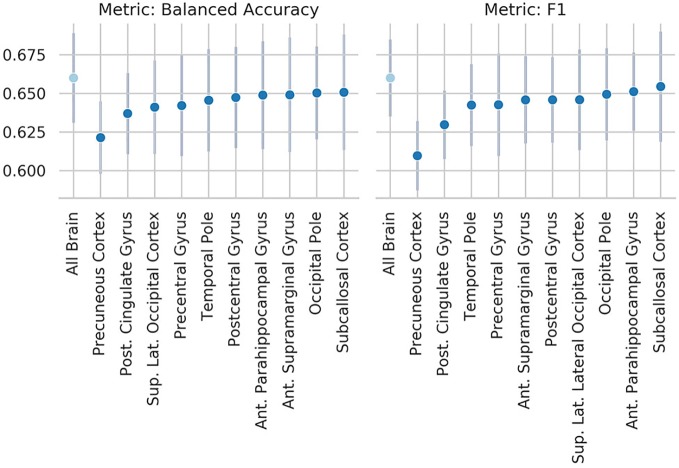
Results of the occlusion experiment for the regional homogeneity (ReHo) linear-support vector machine (SVM), balanced accuracy, and f1 are reported as mean and standard deviation across five folds. “All brain” indicates the results obtained by the original model, when all the region of interests (ROIs) are included. ROIs have been identified using the Harvard-Oxford atlas (threshold at 25 and downsampled at 4 mm). The ROI names indicate the results when the named ROI is masked from the brain volume, therefore the drop from the “all brain” result is a suggestion of how much the voxels contained in the ROI contributed to the original results. Only the 10 ROIs that contributed the most are reported. Abbreviations are as in **Figure 5**.

To explore which brain regions were maximally contributing to the classification results, we performed an occlusion experiment on the most successful summary measure: the ReHo, both as input to the SM-model and the SVM. Mean and standard deviation across folds of the 10 regions which occlusion drove the steeper drop in performance are reported in **Figures 5** (SM-model) and **Figure 6** (SVM). Interestingly, the occlusion experiments highlighted many common areas which masking caused a sensible corruption of classification performance. The precuneus was the region maximally driving the results in all the experiments.

## Discussion

We have proposed a machine learning solution for using rs-fMRI that does not compromise its spatial properties. And we presented an empirical analysis of how the choice of summary of the temporal dimension *via* various statistical measures can impact the performance of a 3D convolutional neural network in classifying ASD subjects from the control subjects. We considered nine different measures and used them as inputs in a 3D-CNN model either as i) independent inputs to different 3D-CNNs (SM-models), ii) an ensemble of results from the nine independent 3D-CNN models to one output (MM-ensemble), or iii) a combined nine channel 3D-CNN model that used each measure as a channel.

Our analyses suggest that using a single summary measure is often suboptimal for training 3D-CNNs, and more accurate predictions can be achieved with an ensemble approach, even in a heterogeneous dataset such as ABIDE I+II. Each single summary measure extracts specific information from the rs-fMRI data, capturing local or global aspects of the connectivity of the voxels in the volumes. Even though different modalities may result in similar accuracy performance, the trained models may contain distinct information. This is confirmed by the correlation matrix of predictions (**Figure 4**). We calculated the agreement between all the SM-models and each SM-model with the MM-ensemble. Combining models from different modalities enhances the performance and creating an ensemble of these measures at the last stage of outcome prediction, as done by the MM-ensemble model, seems to take advantage of the multiple representation of the data without being affected by the increased noise.

Our MM-model's average performance was below 60%, which is 4% less than the MM-ensemble and less than many of the SM models. Here, summary measures that are not conveying information about the classification task but who were pooled together with informative summary measures potentially increased the noise of the input data and therefore complicated the classification task for the model.

The concept of transforming weak learning algorithms into stronger learners by ensembling them has been proven successful in a number of computer vision tasks (31, 32). In the study of Khosla and colleagues, (16), the authors affirmed to have overcome the limitation of traditional machine learning models for connectomes that rely on region-based summary statistics by ensembling the different atlases into a single model, with a small gain in accuracy, for a final performance of 72.3%.

Even though the MM-ensemble approach resulted in the best performance on the ASD classification, also some of the summary measures reached a good classification performance. In particular ReHo resulted in the best accuracy performance across the SM-models. However, this knowledge is only available after performing the experiments and thus difficult to anticipate when choosing a particular summary measure. The MM-ensemble approach seems to benefit from the performance of ReHo without the need to select this measure *a priori*. Differences in ReHo between ASD and CON have indeed been reported in the literature. For example, the pericalcarine visual cortex was found to be locally hyperconnected in the ASD compared to CON (33), and subjects with ASD have right dominant ReHo alterations of resting-state brain activity, i.e., areas known to exhibit abnormal stimulus or task related functionality (34). Decreased ReHo in the ASD group compared to the CON group was found in bilateral middle and superior frontal gyri, left superior parietal lobule, and right precuneus. Increased ReHo in the ASD group compared to the CON group was found in bilateral middle temporal and right parahippocampal gyri. The authors also report that the ReHo in the precuneus correlated with the autistic trait score. Jiang and colleagues found enhanced local connectivity in the middle frontal cortex, left precuneus, and right superior temporal sulcus, and reduced local connectivity in the right insular cortex using ReHo (35).

These results are consistent with our occlusion experiments, which identified the precuneus and occipital cortex as the regions of the brain that had the most influence on the ReHo-SM-model and the ReHo-SVM outputs. The occlusion procedure used here has the advantage to be easily interpretable and to open a window to the information used by the models to perform the given task, but it is merely descriptive and no strong conclusions can be drawn from its results. The drop in performance after occluding the precuneus is of only ~3 percentage points, suggesting that the algorithms are more likely identifying patterns spanning more than one single brain region.

The studies mentioned used small groups to detect differences in ReHo between groups, and our findings on the large ABIDE I+II dataset suggest that ReHo is indeed a sensitive measure for detecting cortical abnormalities in autism.

The field of neuroimaging is benefiting from the development of deep learning techniques and a growing number of studies have applied deep learning for classification of ASD using the ABIDE dataset. Unfortunately, comparison between results is made hard by the heterogeneity of data preprocessing, data selection, and model selection procedures. We followed the data selection procedure described in (16). They obtained a top accuracy of 72.3% by summarizing the temporal dimension of the data in connectome matrices calculated averaging stochastically determined regions of interest. Their model therefore outperformed our best model, the MM-ensemble, which reached an average balanced accuracy of 64 and 66% F1-score in the K-fold CV procedure (but reduced performance in leave-site-out procedure, see **Table 3**). While they used data from ABIDE-I for training and test on ABIDE-II, we trained all our models on a mix of data from both ABIDE-I+II. Since the increased heterogeneity of our training dataset could possibly explain the decrease in performance, we repeated the analyses of the 3D-CNN models, training the models on the data from ABIDE-I and testing the performance on ABIDE-II (the results are reported in **Supplementary Table S3**). Our approach of summarizing the rs-fMRI data on the temporal domain still showed lower performances compared with (16). These differences might be because Khosla et al., used the connectivity between ROIs and not the statistics from individual voxels to perform the classification, thus hinting at the possibility that connectivity patterns across the brain contain crucial information for the classification between ASD and CON.

The 3D CNN model we employed in our series of experiments was inspired by the one described in Khosla and colleagues but we decrease the number of CNN layers from four to two. The reason behind this choice lies in the fact that, contrary to the original model, we do not apply regularization techniques to our 3D-CNN. Our model has a total of 257,585 trainable parameters. This number of free parameters is “small” if compared to some state of art networks for computer vision. As comparison, the ResNet 50 has over 23 millions trainable parameters, but the number of brain volumes available for training is also critically smaller than the number of images a ResNet50 was trained on (e.g., ImageNet has > 14 millions of images). The number of trainable parameters in our model is suitable for the number of training examples, and a larger network will be more prone to overfitting. Indeed, when our 3D-CNN model is left training for enough time (approximately 50 epochs), it is able to reach 100% accuracy on the training set at the expense of generalizability to new data: a clear indication of overfitting. For these reasons described, it is unlikely that the 3D-CNN model was too shallow or not trained enough.

We built our model on the example of another model in the literature, which has proven successful on the same task of classifying ASD from CON. The original model was trained on different features then ours. The relatively low classification accuracy that even our best model obtained might be a consequence of this choice: the parameters that made the original model successful did not generalize to our input data. Indeed we performed a non-systematic parameter and hyper-parameter search and carried out the full experiments on the best configuration. Unfortunately, the numbers of adjustable parameters in CNN models are extremely high, and it would be computationally prohibitive to carry out a full systematic search.

The ABIDE dataset is composed of distinct datasets collected by different institutions. The data collected vary in terms of demographics of the participants, scanning hardware, and sequences for data collection, therefore the images vary in image quality and resolution. Differences in resolution do not present a concern, because all the images were resampled to the same 4x4x4 mm3 voxel size in MNI space. Differences in sample composition and data acquisition contribute considerably to the heterogeneity of the data, which has been identified as one of the most important limitations of the ABIDE dataset, and therefore might explain the low accuracy achieved (36).

In general, heterogeneity of the dataset has been pointed out by many as a limitation in performing ML on neuroimaging data (37). Classification accuracy drops significantly in larger population samples and especially when the data are aggregated from different sites (36).

Another possibility is that the time domain of rs-fMRI data contains properties that get lost when the summarizing procedures are applied. Correlation and its derivative (like our summary measures) are first-order transformation, which does not account for higher-order interactions between time courses. In previous work of our group, we maintained the time dimension while summarizing the spatial dimension using ROIs (Harvard Oxford atlas). This approach obtained an accuracy of 68% using a simple 1D-CNN model on the ABIDE I+II dataset (3).

Another interesting finding is that the linear-SVM performed as well, and in certain instances better, than the 3D-CNN models. He et al., (8), have found that SVM do as well as 3D-CNNs in other tasks as well. We hypothesize that in our task, there was no apparent underlying structure in these 3D summary measures that could be exploited. A linear-SVM can be thought of as a fully connected neural network with non-linear activation function (sign function). Our 3D-CNN included also a fully connected last layer that can again be thought of as an SVM on the representations learnt by preceding convolutional layers. The fact that the 3D-CNN architecture could not outperform the linear-SVM suggests that either there were no low-dimensional patterns that capture the essence of the disorder in these summary measures, or that the amount of data is insufficient for the 3D-CNN to learn interesting structures. The amount of data available might not be sufficient to leverage the ability of CNNs to detect patterns. CNNs are highly flexible models that have been developed in the context of “big data” settings. The sample size in our experiments is large but probably not large enough to take full advantage of CNN models. This could explain accuracies similar to those of much less flexible linear-SVM models.

We have shown that simple temporal transformation can lead to accuracies comparable to state-of-the-art for a complex task like classifying ASD from control subjects. But we also found that there is not much advantage in using a 3D-CNN architecture for this task. We have, including in our previous studies and this, shown various ways of reducing the dimension of the rs-fMRI signal before feeding it into a machine learning algorithm. In the future, we plan to utilize the full 4D structure of the rs-fMRI without compromising the resolution in either time or space. This can be achieved for example by exploiting larger datasets like the UK Biobank^6^ to learn representations for rs-fMRI signals which can then be used in small-sample psychiatric datasets like ABIDE.

## Data Availability Statement

The datasets generated and the 3D convolutional models used for this study are available on request to the corresponding author.

## Author Contributions

SG and RT contributed equally to the paper and are co-first authors. SG performed the experiments and drafted the first version of the manuscript. RT designed the architecture of the models and contributed to the manuscript. RT and GW conceived the problem. LC, AA-G, and PZ contributed to designing the experiments and preprocessing of neuroimaging data. All authors contributed to the final manuscript.

## Funding

This work was supported by the Netherlands Organization for Scientific research (NWO/ZonMw Vidi 016.156.318).

## Conflict of Interest

GW received funding from Philips Research for another research project.

The remaining authors declare that the research was conducted in the absence of any commercial or financial relationships that could be construed as a potential conflict of interest.

## Footnotes

1 https://github.com/galloselene/TempStats_3D-CNN.git

2 http://fcon_1000.projects.nitrc.org/indi/abide/

3 http://preprocessed-connectomes-project.org/abide/

4 https://fcp-indi.github.io/docs/user/quick.html

5 https://scikit-learn.org/

6 https://www.ukbiobank.ac.uk/
