# Supplementary material for: Classifying Autism Spectrum Disorder Using the Temporal Statistics of Resting-State Functional MRI Data With 3D Convolutional Neural Networks
Source: Front Psychiatry. 2020 May 15;11:440. doi: 10.3389/fpsyt.2020.00440 (PMC7242627; doi:10.3389/fpsyt.2020.00440)
Supplement: Supplementary file 1 [file Table_1.docx]

|  | **Site used as *test*** | **Accuracy** | **F1-Score** |
| --- | --- | --- | --- |
| alff | NYU | 0,66 | 0,66 |
| alff | ABIDEII-KKI_1 | 0,47 | 0,64 |
| alff | UM_1 | 0,61 | 0,61 |
| alff | UCLA_1 | 0,62 | 0,59 |
| alff | ABIDEII-OHSU_1 | 0,57 | 0,63 |
| autocorr | NYU | 0,51 | 0,28 |
| autocorr | ABIDEII-KKI_1 | 0,50 | 0,67 |
| autocorr | UM_1 | 0,52 | 0,42 |
| autocorr | UCLA_1 | 0,50 | 0,23 |
| autocorr | ABIDEII-OHSU_1 | 0,50 | 0,52 |
| degree centrality | NYU | 0,58 | 0,59 |
| degree centrality | ABIDEII-KKI_1 | 0,59 | 0,57 |
| degree centrality | UM_1 | 0,62 | 0,61 |
| degree centrality | UCLA_1 | 0,52 | 0,54 |
| degree centrality | ABIDEII-OHSU_1 | 0,48 | 0,50 |
| eigenvector centrality | NYU | 0,55 | 0,55 |
| eigenvector centrality | ABIDEII-KKI_1 | 0,55 | 0,50 |
| eigenvector centrality | UM_1 | 0,47 | 0,41 |
| eigenvector centrality | UCLA_1 | 0,51 | 0,49 |
| eigenvector centrality | ABIDEII-OHSU_1 | 0,49 | 0,51 |
| entropy | NYU | 0,51 | 0,46 |
| entropy | ABIDEII-KKI_1 | 0,50 | 0,67 |
| entropy | UM_1 | 0,52 | 0,50 |
| entropy | UCLA_1 | 0,48 | 0,23 |
| entropy | ABIDEII-OHSU_1 | 0,52 | 0,57 |
| falff | NYU | 0,52 | 0,53 |
| falff | ABIDEII-KKI_1 | 0,53 | 0,55 |
| falff | UM_1 | 0,57 | 0,56 |
| falff | UCLA_1 | 0,52 | 0,54 |
| falff | ABIDEII-OHSU_1 | 0,55 | 0,61 |
| lfcd | NYU | 0,60 | 0,61 |
| lfcd | ABIDEII-KKI_1 | 0,51 | 0,63 |
| lfcd | UM_1 | 0,58 | 0,56 |
| lfcd | UCLA_1 | 0,55 | 0,54 |
| lfcd | ABIDEII-OHSU_1 | 0,44 | 0,45 |
| reho | NYU | 0,69 | 0,70 |
| reho | ABIDEII-KKI_1 | 0,56 | 0,66 |
| reho | UM_1 | 0,60 | 0,59 |
| reho | UCLA_1 | 0,59 | 0,58 |
| reho | ABIDEII-OHSU_1 | 0,64 | 0,65 |
| vmhc | NYU | 0,50 | 0,36 |
| vmhc | ABIDEII-KKI_1 | 0,55 | 0,67 |
| vmhc | UM_1 | 0,60 | 0,60 |
| vmhc | UCLA_1 | 0,63 | 0,65 |
| vmhc | ABIDEII-OHSU_1 | 0,51 | 0,55 |
| MM Ensemble | NYU | 0,63 | 0,64 |
| MM Ensemble | ABIDEII-KKI_1 | 0,53 | 0,68 |
| MM Ensemble | UM_1 | 0,54 | 0,52 |
| MM Ensemble | UCLA_1 | 0,55 | 0,51 |
| MM Ensemble | ABIDEII-OHSU_1 | 0,54 | 0,60 |
| MM Model | NYU | 0,67 | 0,68 |
| MM Model | ABIDEII-KKI_1 | 0,51 | 0,66 |
| MM Model | UM_1 | 0,54 | 0,44 |
| MM Model | UCLA_1 | 0,56 | 0,53 |
| MM Model | ABIDEII-OHSU_1 | 0,52 | 0,55 |

**Supplementary Table S1.** Performance evaluated as balanced accuracy (Accuracy) and F1-Score obtained for 3D CNN with leave-site-out CV on each test-site.
