# Supplementary material for: Classifying Autism Spectrum Disorder Using the Temporal Statistics of Resting-State Functional MRI Data With 3D Convolutional Neural Networks
Source: Front Psychiatry. 2020 May 15;11:440. doi: 10.3389/fpsyt.2020.00440 (PMC7242627; doi:10.3389/fpsyt.2020.00440)
Supplement: Supplementary file 3 [file Table_3.docx]

|  | **Accuracy** | **F1-Score** |
| --- | --- | --- |
| reho | **0,60** | **0,60** |
| vmhc | 0,58 | 0,60 |
| falff | 0,55 | 0,55 |
| degree centrality | 0,54 | 0,55 |
| alff | 0,54 | 0,55 |
| lfcd | 0,54 | 0,55 |
| eigenvector centrality | 0,52 | 0,52 |
| entropy | 0,49 | 0,47 |
| autocorr | 0,47 | 0,43 |
| MM Ensemble | 0,56 | 0,58 |
| MM Model | 0,55 | 0,55 |

**Supplementary Table S3.** Performance evaluated as balanced accuracy (Accuracy) and F1-Score obtained for 3D-CNN approach when trained on Abide I (N ASD/CON = 324/619) and tested on Abide II (N ASD/CON= 226/389).
